# Advancements and Future Prospects in Molecular Targeted and siRNA Therapies for Chronic Myeloid Leukemia

**DOI:** 10.3390/biom14060644

**Published:** 2024-05-30

**Authors:** Vera Vysochinskaya, Olesya Dovbysh, Andrey Gorshkov, Alexandra Brodskaia, Michael Dubina, Andrey Vasin, Yana Zabrodskaya

**Affiliations:** 1 Institute of Biomedical Systems and Biotechnology, Peter the Great Saint Petersburg Polytechnic University, 29 Ulitsa Polytechnicheskaya, 194064 St. Petersburg, Russiayana@zabrodskaya.net (Y.Z.); 2 Smorodintsev Research Institute of Influenza, Russian Ministry of Health, 15/17 Ulitsa Prof. Popova, 197376 St. Petersburg, Russia; 3 Almazov National Research Centre, Akkuratova str. 2, 197341 St. Petersburg, Russia; 4 Russian Academy of Sciences, 14 Leninskiy pr., 119991 Moscow, Russia

**Keywords:** chronic myeloid leukemia, BCR–ABL1, tyrosine kinase inhibitors, small interfering RNA

## Abstract

Chronic myeloid leukemia (CML) is an oncological myeloproliferative disorder that accounts for 15 to 20% of all adult leukemia cases. The molecular basis of this disease lies in the formation of a chimeric oncogene BCR–ABL1. The protein product of this gene, p210 BCR–ABL1, exhibits abnormally high constitutive tyrosine kinase activity. Over recent decades, several targeted tyrosine kinase inhibitors (TKIs) directed against BCR–ABL1 have been developed and introduced into clinical practice. These inhibitors suppress BCR–ABL1 activity through various mechanisms. Furthermore, the advent of RNA interference technology has enabled the highly specific inhibition of BCR–ABL1 transcript expression using small interfering RNA (siRNA). This experimental evidence opens avenues for the development of a novel therapeutic strategy for CML, termed siRNA therapy. The review delves into molecular genetic mechanisms underlying the pathogenesis of CML, challenges in CML therapy, potential molecular targets for drug development, and the latest results from the application of siRNAs in in vitro and in vivo CML models.

## 1. Introduction

Chronic myeloid leukemia (CML) is a form of myeloproliferative disorder characterized by the malignant transformation of early hematopoietic stem cells. This process results in the clonal proliferation of myeloid cells, which retain their capacity for differentiation and maturation, accounting for approximately 15 to 20% of adult leukemia cases. The pathogenesis of CML is attributed to a genetic abnormality known as the Philadelphia chromosome (Ph), delineated by Nowell and Hungerford in 1960 [1]. This chromosomal abnormality arises from the reciprocal translocation between chromosomes 22 and 9, denoted as t(9:22) (q34; q11) [2]. Such a translocation fuses the *breakpoint cluster region* (*BCR*) gene from chromosome 22 with the *Abelson proto-oncogene* (*ABL1*) from chromosome 9, culminating in the creation of the chimeric oncogene *BCR–ABL1* on chromosome 22, which encodes the chimeric oncogenic protein p210 BCR–ABL1 (210 kDa). The constitutive tyrosine kinase activity of this protein is responsible in the malignant transformation of bone marrow stem cells, driving the pathogenesis of CML [3,4,5,6].

CML progresses through three stages: the chronic stage, the accelerated stage, and the blast crisis stage. The chronic stage features an increase in the number of myeloid cells, which retain the ability to differentiate and function. The accelerated stage, which lasts from a few weeks to several years, is characterized by the emergence of immature cells in the bloodstream. These cells replace the differentiated cells from the myeloid stem cell lineage of hematopoiesis. The blast crisis stage is dominated by immature blast cells and associated with a median survival time of just several months for patients. The clinical presentation of the disease deteriorates markedly as CML progresses, starting with mild symptoms during the chronic stage. In both the accelerated and blast crisis stages, the proliferation of the tumor cell clone and disruption of normal hematopoietic stem cell function leads to severe thrombocytopenia, characterized by low platelet counts, which results in hemorrhagic complications and cancer-induced toxicity [7].

Molecular studies of CML pathogenesis have laid the foundation for the development of targeted therapies. Consequently, BCR–ABL1 tyrosine kinase inhibitors (TKIs), a type of small-molecule drug, were developed, showcasing the translation of fundamental knowledge to clinical practice. The introduction of TKIs has revolutionized the management of CML, turning a once fatal disease into one where the life expectancy of patients is nearing that of the general population [8]. Despite the remarkable success of TKIs in clinical settings, the challenge of developing resistance to TKI therapy persists. For 5% of patients, disease progression to the blast crisis stage is possible against the backdrop of treatment. Resistance to TKI therapy, alongside the increasing costs of treatment and a substantial patient population, underscores the need for developing new treatment modalities for CML [9].

Based on the understanding of the role of specific genes in the development of malignant tumors, a new approach to drug development has emerged, targeting these genes directly. One of the most promising methods of gene therapy is antisense therapy, which employs short antisense oligonucleotides, primarily small interfering RNA (siRNAs). siRNAs interact with specific mRNA sequences of the target genes, suppressing gene expression through RNA interference [10,11]. The utilization of siRNA technology offers the potential for the development of fundamentally new and effective treatment methods for CML. Additionally, siRNAs can target both *BCR–ABL1* and transcripts from a number of other critical genes involved in the progression of CML.

Our review article delves into the molecular mechanisms behind the development of CML and explores the current challenges in treating CML with targeted therapies. We elaborate on various pharmacological strategies for inhibiting BCR–ABL1 and provide a succinct overview of clinical experiences with these drugs. Furthermore, we provide a detailed analysis of the results from employing siRNA in both in vitro and in vivo models of CML, highlighting its potential as an innovative treatment strategy for this formidable disease.

## 2. The Protein p210 BCR–ABL1 Is a Key Player in the Pathogenesis of CML

The pathogenesis of CML is linked to the emergence of a pathological protein, the chimeric BCR–ABL1 protein, in myeloid cells. This protein, originating from the Philadelphia chromosome, combines domains from both BCR and ABL1 proteins. The *BCR* gene, initially identified within the *BCR–ABL1* oncogene, has been found in various fusion variants with other genes, leading to chimeric proteins that significantly contribute to the development of several oncohematological diseases [12]. Despite being a common partner in gene fusions during carcinogenesis, the native function of the *BCR* gene remains elusive [13]. The *BCR* gene encodes a protein with a molecular weight of 160 kDa, featuring several domains:

- The N-terminal region possesses a coiled-coil oligomerization domain (CC) implicated in binding to proteins containing the Src-homology 2 domain.
- A serine/threonine kinase domain capable of phosphorylating tyrosine residues, including tyrosine 117 (Y177).
- A segment homologous to the Rho guanine nucleotide exchange factor (Rho-GEF).
- A calcium-binding domain (CaLB).
- A region resembling the Rac GTPase-activating protein (Rac-GAP).

**Figure 1:**
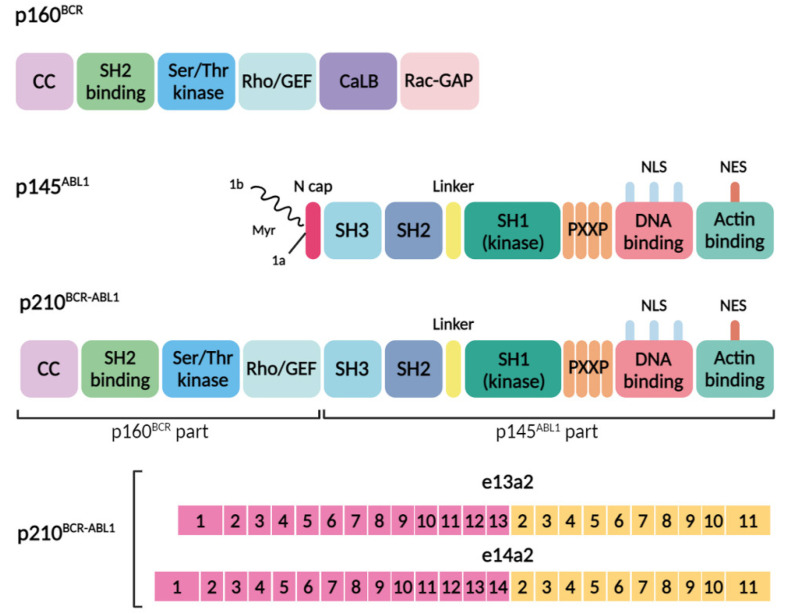
Domain organization of the BCR, ABL1, and BCR–ABL1 proteins and common genomic breakpoints within the *BCR* and *ABL1* genes, along with the resultant transcript types and proteins. The domain organization of p160^BCR^ includes the coiled-coil oligomerization domain (CC), Src-homology 2 domain (SH2 binding), serine/threonine kinase domain (Ser/Thr kinase), segment homologous to the Rho guanine nucleotide exchange factor (Rho-GEF), calcium-binding domain (CaLB), and a region resembling the Rac GTPase-activating protein (Rac-GAP). The domain organization of p145^ABL1^ consists of the N-cap (1a and 1 b isoforms of the protein, with isoform 1b undergoing myristoylation (Myr) of the N-terminal glycine), three homologous protein domains: SH3, SH2, SH1, a proline-rich (PXXP) motif, nuclear localization signals (NLS), a nuclear export signal (NES), a DNA-binding region, and an actin-binding region. The p210^BCR-ABL1^ fusion oncoprotein consists of domains from the BCR (p160^BCR^ part) and ABL1 (p145^ABL1^ part) proteins, and the fusion results in constitutive activation of the tyrosine kinase. The *BCR-ABL1* fusion gene consists of the 5′ end of the *BCR* gene (pink) and the 3′ end of the *ABL1* gene (yellow). The breakpoints of the translocation usually involve intron 13 or 14 of *BCR* (p210^BCR-ABL1^ with an e13a2 junction or p210^BCR-ABL1^ with an e14a2 junction).

The CC domain is known to play an important role in the kinase activity of its fusion partners (Figure 1) [14]. The *BCR* gene expression is observed in cells at the earliest stages of myeloid differentiation and significantly diminishes as cells mature. Research suggests that BCR acts as a negative regulator of cell proliferation and oncogenic transformation. It interacts with AF-6, a member of the Ras Association Family 6, forming a complex believed to inhibit Ras-mediated signaling at intercellular junctions [15].

The ABL1 protein, with a molecular weight of approximately 145 kDa, encompasses several crucial functional domains. In isoform 1b of the protein, the N-terminal glycine undergoes myristoylation—a post-translational modification that attaches myristate, a saturated fatty acid with fourteen carbon atoms, to the glycine. The protein is composed of three homologous protein domains: SH1, SH2, and SH3. Notably, the SH3 domain, characterized by tyrosine-kinase activity, includes four proline-rich regions essential for binding to other proteins. Moreover, it is equipped with three nuclear localization signals (NLS) that facilitate its entry into the cell and the nucleus, alongside one nuclear export signal (NES) that enables its exit. The protein also features a DNA-binding region and an actin-binding region (Figure 1), both crucial for its involvement in various cellular processes [16].

Based on crystallographic data, the catalytic domain of the p145 ABL1 protein is composed of two lobes joined by a flexible hinge. The upper, N-terminal lobe features five antiparallel beta sheets, a single short alpha-helix, and is tasked with ATP binding. The lower, C-terminal lobe is primarily helical. Located at the juncture of the two lobes is a slot housing both the ATP-binding site and the catalytic site. In the active state of the enzyme, the activation loop (A-loop) does not obstruct the catalytic center. The C-terminal segment of the A-loop is crucial for substrate binding. The kinase is activated through the phosphorylation of tyrosine at position 393 within the A-loop, which repositions the A-loop, thus facilitating substrate attachment. Conversely, when tyrosine 393 is dephosphorylated, it forms a hydrogen bond with asparagine 363 in the inactive enzyme conformation, precluding substrate interaction [17,18].

The non-receptor protein tyrosine kinase p145 ABL, is pivotal in hematopoietic processes. Its SH1 domain acts as a tyrosine kinase, catalyzing the phosphorylation of tyrosine-containing proteins to relay proliferative signals within the cell. The SH2 and SH3 domains, also part of the tyrosine kinase domains, govern the interaction between p145 ABL and other proteins, notably integrin and actin [16]. Additionally, the SH3 domain is essential for regulating protein kinase activity, with its deletion activating the kinase function of the protein in a manner akin to that observed in BCR–ABL1 [19]. Positioned at the C-terminus, the actin-binding domain of p145 ABL significantly influences cytoskeleton organization and cell–cell adhesion. Broadly speaking, p145 ABL orchestrates the control of the cell cycle, responses to genotoxic stress, and the transmission of signals from the cellular milieu via integrin interaction. This protein amalgamates signals from diverse external and internal stimuli, thereby managing cell cycle dynamics and apoptosis [20,21].

The formation of a Philadelphia chromosome through reciprocal translocation is invariably linked to the presence of the *BCR–ABL1* gene. Nevertheless, the molecular characteristics of this event can vary depending on the precise locations where the genes break. Variants of the *BCR–ABL1* chimeric oncogene differ from each other in the breakpoint locations during the translocation, t(9;22) [22]. In CML, the *BCR* gene most frequently breaks at introns 13 and 14 (e13 and e14), which constitute the major breakpoint cluster region (M-bcr). Meanwhile, breaks within the *ABL1* gene commonly occur in the 5′ extended region upstream of exon a2 (beyond 300 base pairs). The vast majority of CML patients express one or two transcripts, e13a2 (b2a2) and e14a2 (b3a2), which vary by the inclusion of one *BCR* exon. These transcripts translate into protein products with a molecular weight of approximately 210 kDa (p210), representing the predominant protein form in CML patients (Figure 1). Other chimeric transcripts resulting from alternative breakpoints are less frequently observed [23].

As a result of this reciprocal translocation, a chimeric protein, p210 BCR–ABL1, is formed. This protein encompasses the first four domains from the BCR sequence, while the remainder of the protein includes all the domains from the ABL sequence, except for the N-terminus of the SH3 domain (Figure 1). The loss of its N-terminal portion upon fusion with BCR, leads to the deregulation of ABL1’s tyrosine kinase activity. Crucially, the myristoylation of the N-cap region of the ABL protein, vital for autoregulation, binds to the hydrophobic pocket in the kinase’s C-terminal lobe. This binding prompts changes in the conformation of the C-terminal helix, necessary for the formation of an SH3–SH2 clamp to keep the kinase inactive. The absence of this site, combined with the incorporation of the BCR sequence including the oligomerization domain, results in the loss of physiological control over kinase activity [21]. Consequently, a plethora of substrates are phosphorylated, impacting signaling pathways such as GRB2/GAB2, CRKL, JAK/STAT, and MAPK/PI3K/AKT [24,25,26]. The disruption of ABL1 tyrosine kinase’s normal function leads to malignant transformation processes, including the disruption of cellular adhesion to the bone marrow stroma and the extracellular matrix, a constitutive signal for cell proliferation, the downregulation of cellular differentiation, and a reduction in apoptosis. These mechanisms underlie the majority of phenotypic manifestations observed in CML [26,27].

## 3. Strategies for Inhibiting the Chimeric Protein BCR–ABL1

**Figure 2:**
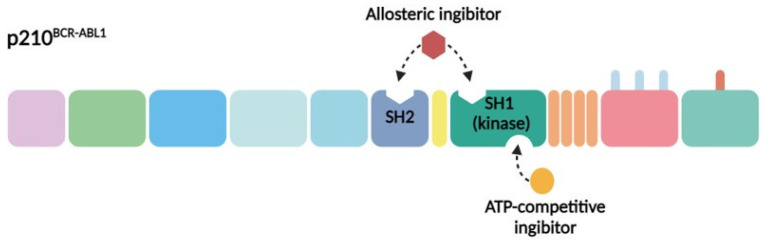
Strategies for pharmacological inhibition of BCR–ABL1. The SH1 kinase domain (green) and SH2 domain (blue) are both vital targets for pharmacological inhibition. The ATP-competitive inhibitors, such as imatinib, dasatinib, bosutinib, and nilotinib—depicted in yellow—target these domains. Allosteric inhibitors, represented in burgundy, which simulate myristate binding to the hydrophobic pocket on the C-lobe, like asciminib, are also under exploration. Moreover, the use of anti-SH2 monobodies is being investigated.

The primary contemporary strategy for treating CML patients involves the pharmacological inhibition of BCR–ABL1. Given the structural and functional attributes of this chimeric protein and its pivotal role in CML pathogenesis, two principal approaches have been devised for its inhibition. The initial method entails the design of molecules that compete with ATP (adenosine triphosphate) for binding to the ATP-binding pocket of BCR–ABL1, thus impeding its tyrosine kinase activity. This strategy involves the creation of molecules that latch onto specific regions on the protein, preventing the kinase from operating effectively. The second method focuses on the development of allosteric inhibitors that attach to parts of the protein other than the ATP-binding site. These inhibitors also impede kinase activity by targeting distinct sites crucial for the regulation of kinase function (Figure 2) [14]. The BCR–ABL1 inhibitors currently used in clinical practice are presented in Table 1.

### 3.1. ATP-Competitive Inhibitors of BCR–ABL1

In the 20th century, allogeneic hematopoietic stem cell transplantation (allo-HSCT) represented the sole curative treatment option for CML, capable of inducing complete remission. Following the elucidation of the ABL protein’s crystal structure, drug development efforts have concentrated on designing molecules that target the ATP-binding site of the kinase domain, aiming to abate the aberrant tyrosine kinase activity of the protein. This endeavor led to the synthesis of STI 571 (Imatinib Mesylate or Gleevec), a 2-phenylaminopyrimidine derivative tyrosine kinase inhibitor by Novartis [28].

The advent of imatinib ushered in a new era of targeted cancer therapy. In vitro studies utilizing cell cultures from CML patients demonstrated that Gleevec (imatinib) markedly inhibits the proliferation of the Ph-positive cells, while sparing Ph-negative cells. X-ray crystallography insights revealed that imatinib snugly fits into the BCR–ABL1 protein’s ATP binding pocket, outcompeting ATP and thereby incapacitating the kinase. Consequently, the signal transduction pathway that promotes tumor cell growth is obstructed [28]. Despite imatinib’s impressive clinical outcomes, the quest for an ultimate cure for CML persists.

Although highly efficacious, primary or secondary resistance to TKI therapy emerges in approximately 20% to 30% of cases [29,30]. Resistance to TKIs stems from a multitude of intertwined factors, including the treatment regimen, TKI pharmacodynamics, and genetic alterations within the BCR–ABL1 kinase domain [31]. The predominant mechanism of resistance involves mutations across various sites of the *BCR–ABL1* gene encoding the tyrosine kinase’s catalytic domain, particularly within the ATP-binding P-loop and the activating loop (A-loop). The incidence of these mutations varies, influenced by resistance criteria, detection methodologies, and disease stage. Such kinase domain point mutations induce structural alterations that hinder the drug’s attachment to the tyrosine kinase pocket, precluding ATP binding through steric hindrance [32]. Notably, mutations in the P-loop, especially the T315I substitution (where threonine is replaced with isoleucine), pose substantial challenges for TKIs efficacy as this region encompasses the ATP-binding site [33].

Beyond mutations, other elements such as certain genetic variations and dosing strategies contribute to imatinib resistance [34,35,36,37]. To overcome these hurdles, several second-generation TKIs have been developed, including nilotinib (Tasigna), dasatinib (Sprycel) and bosutinib (Bosulif), boasting a higher binding efficiency to BCR-ABL1 than imatinib. Nilotinib binds the inactive conformation of BCR–ABL1, whereas dasatinib, suppresses kinase activity across both inactive and active forms of BCR–ABL1. Bosutinib, a dual inhibitor, acts against both BCR–ABL1 and the Src kinase with a potency 10–100 times that of imatinib. Nevertheless, these TKIs fall short of overcoming the T315I resistance mutation [38,39]. Ponatinib remains the sole FDA-approved medication effective against the T315I mutation in CML. However, its broad activity spectrum compromises its safety profile, potentially leading to severe side effects [40,41].

The employment of second-generation TKIs often enables the circumvention of resistance and intolerance issues associated with imatinib in many patients. Nevertheless, there are instances where even these novel drugs prove ineffective. In situations where resistance to second-line TKI therapies is observed, or when the T315I mutation is detected, as well as for patients with CML in the accelerated phase or blast crisis, allo-HSCT is advocated. The feasibility of such transplantation, however, is significantly constrained by the risks posed by coexisting pathologies and the scarcity of compatible donors [9].

### 3.2. Allosteric Inhibitors of BCR–ABL1

Several allosteric sites on the BCR–ABL1 protein present promising targets for drug development. Key to this discussion is the role of myristoyl groups binding to myristate pockets, a process that triggers critical conformational changes in the kinase domain’s C-terminal helix. This action is pivotal for initiating an SH2–SH3 clamp, thereby rendering kinases inactive. Utilizing this mechanism, researchers have developed compounds like GNF-2 and GNF-5, designed to simulate the impact of myristate binding [42]. Upon binding to the BCR–ABL1, these compounds promote conformational changes that deactivate the protein. However, mutations at the myristoyl binding site, such as C464Y, P465S, and V506L, have been identified as potential sources of resistance to these treatments. GNF-2’s clinical trials were halted due to its inefficacy against the T315I mutation. GNF-5, rebranded as ABL001, showed promise in clinical trials, leading to its FDA approval in 2021 for Ph positive (Ph+) CML patients in the chronic phase previously treated with two or more TKIs, including those with the T315I mutation. Employing a dual-approach strategy that combines ATP-competitive and allosteric BCR–ABL1 inhibitors from the outset, might optimize resistance prevention [43,44,45].

The SH2 kinase domain has emerged as a vital regulatory area that amplifies kinase activity, marking it as an additional therapeutic target [46]. “Monobodies”, proteins modeled after the type III fibronectin domain, are undergoing research for their ability to bind intracellular targets with significant affinity and specificity, potentially outperforming small molecule inhibitors. Notably, their reduced size facilitates cell penetration for intracellular targeting, unlike traditional monoclonal antibodies. Monobodies targeting the SH2 of BCR–ABL1 have demonstrated efficacy in hampering kinase activity within CML cells both in vitro and in ex vivo conditions, leading to noteworthy cell apoptosis. Delivery of monobody-based therapeutics has been achieved through lentiviral transfection. Despite its considerable potential, the translation of this approach to in vivo application necessitates the development of efficient and safe intracellular delivery methods to advance these drugs to clinical practice [47].

### 3.3. Inhibition of the Chimeric Oncogene BCR–ABL1 Using siRNA

The inhibition of the chimeric oncogene *BCR–ABL1* using siRNA presents a compelling alternative to conventional TKI therapies, especially in the context of treatment resistance. RNA interference (RNAi) techniques offer a novel approach by specifically targeting and suppressing the expression of chimeric transcripts produced by the *BCR–ABL1* oncogene. The strategic use of siRNAs for cancer therapy carries distinct advantages over traditional small molecule drugs. Unlike these drugs, which bind to the protein’s active site, siRNAs engage with mRNA with high specificity, acting at a molecular level before protein synthesis. This specificity means that siRNA-mediated therapies are potentially less affected by point mutations in nucleotides, which can alter protein conformation and lead to TKI resistance.

Moreover, RNAi technology allows for a broader selection of therapeutic targets, facilitated by the simplicity of siRNA design and production. Given that resistance to the single-agent therapies is a prevalent issue, employing a “cocktail” of siRNAs directed at multiple oncogenic genes could pave the way for effective combination therapy. The application of siRNAs as a novel therapeutic class of drugs for targeted cancer treatment holds significant promise for the field of clinical oncology.

**Figure 3:**
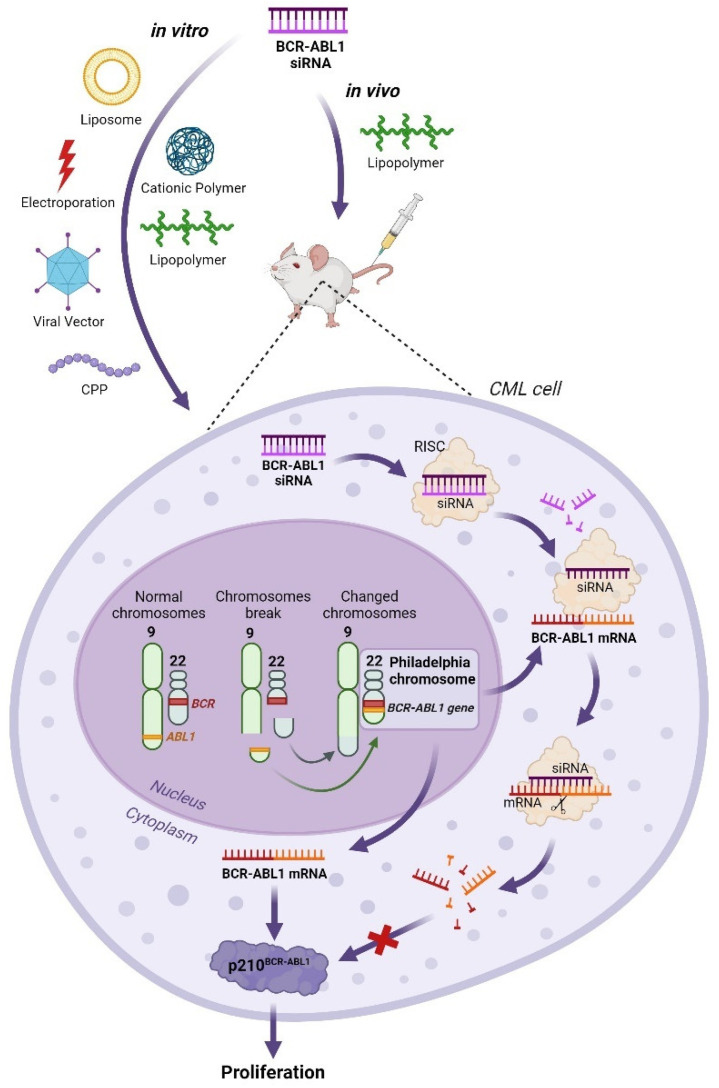
Application of BCR–ABL1 siRNA in in vitro and in vivo CML models. The feasibility of BCR-ABL1 siRNA therapy in CML K562 cells in vitro was investigated using various delivery systems, including liposomes, cationic polymers, lipopolymers, electroporation, viral vectors, and cell-penetrating peptides (CPPs). Lipopolymer nanoparticles were successfully employed for delivering BCR-ABL siRNA in a CML model in vivo. In both cases, the BCR-ABL1 siRNA was delivered to CML cells. In the cell nucleus, after the formation of the Philadelphia chromosome, the *BCR-ABL1* gene is produced. This results in the expression of the p210^BCR-ABL1^ protein, which leads to abnormal cell proliferation. In the presence of BCR-ABL1 siRNA, the BCR-ABL1 mRNA was degraded via the RNA interference mechanism, preventing the formation of the p210^BCR-ABL1^ protein and consequently halting abnormal cell proliferation.

Recent research demonstrates that siRNA can effectively suppress the expression of the *BCR–ABL1* chimeric gene in vitro and in vivo (Figure 3). Various methods, including liposomal delivery [48,49,50,51], electroporation [52,53], viral vector constructs [54], cell-penetrating peptides [51,55,56,57], cationic polymers [58,59], and lipopolymer nanoparticles [60,61] have all been utilized to introduce synthetic anti-*BCR–ABL1* siRNAs into target cells.

The K562 cell line, which harbors the *BCR–ABL1* gene, serves as a prevalent model for in vitro CML research. Studies have indicated that transfecting K562 cells with synthetic BCR–ABL1 siRNA can significantly reduce the half-maximal inhibitory concentration (IC50) of the drug Gleevec by over threefold [49]. This approach has also been shown to suppress the expression of *BCR–ABL1* gene transcripts in cell lines resistant to nilotinib, including those with the clinically significant T315I mutation in the BCR–ABL1 kinase domain. Furthermore, combining BCR–ABL1 siRNA with TKI treatment has been demonstrated to have an additive effect in both sensitive and resistant cell lines, offering new avenues for circumventing resistance in CML therapy [49,62].

The studies mentioned above, focusing on the use of siRNA against *BCR–ABL1* gene transcripts in in vitro cellular models, demonstrate its efficacy in suppressing gene expression. However, the impact of this treatment on leukemic cell proliferation and apoptosis presents a more complex picture. Unlike TKIs, which are known to actively reduce proliferation in CML models, the anti-proliferative effects of BCR–ABL1 siRNA are less consistent, with some studies failing to observe any significant impact. For instance, Scherr et al. reported that siRNAs do not inhibit colony formation of CML cells [63]. The lack of a pronounced antiproliferative effect of synthetic siRNAs could be attributes to their rapid degradation within cells. Moreover, silencing the *BCR–ABL1* gene alone might not sufficiently curb cell proliferation. Nevertheless, some studies [61] have found that the combined application of lipoplexed siRNAs and imatinib can reduce the colony formation in primary cells derived from CML patients. This suggests a variable efficacy of BCR–ABL1 siRNA treatment, potentially pointing to differences in siRNA therapy response or in the effectiveness of siRNA delivery among patients. These findings underscore the importance of identifying additional factors involved in CML pathogenesis, beyond BCR–ABL1 [56].

In research conducted by Remnant et al., BCR–ABL1 siRNA, when delivered using PEI conjugated with cholesterol, notably diminished proliferation and colony formation in the K562 cell line, especially when combined with siRNA targeting the *Kinesin Spindle Protein* (*KSP*), a crucial player in cell proliferation and apoptosis. Similarly, Elmaagacli et al. reported effective inhibition of proliferation and induction of apoptosis with siRNAs against both the *BCR–ABL1* and *Wilms tumor 1* (*WT1*) genes [64]. Another promising approach involved targeting the *Growth Factor Independent-1B* (*GFI1B*) gene, implicated in hematopoietic cells maturation signaling pathways and significantly overexpressed in leukemia cells. Cotransfection with GFI1B and BCR–ABL1 siRNAs markedly reduced viability, proliferation, and apoptosis in both the K562 cell line and primary cell lines from early stage CML patients [65]. Beyond targeting the *BCR–ABL1* gene with siRNA, transcription factors like *STAT3*, *STAT5A*, and *STAT5B*—activated by p210 BCR–ABL1 and involved in the malignant cell transformation process—have also been evaluated. Introducing siRNAs against these factors into K562 cells for 12 days prompted apoptosis in leukemic cells, reinforcing the potential of multi-target siRNA strategies in CML therapy [66].

Recent studies have elucidated the role of abnormal Wnt/β-catenin signaling in the onset of myeloproliferative neoplasms triggered by the *BCR–ABL1* fusion gene in CML mouse models [67]. Furthermore, β-catenin overexpression was observed in the granulocyte and macrophage progenitor cells of CML patients during the blast crisis phase. Crucially, aberrant Wnt/β-catenin signaling has been implicated in the development of resistance to TKI therapies via mechanisms independent of *BCR–ABL1* [68,69]. This suggests that targeting the β-catenin pathway could be a novel therapeutic avenue for CML. In this context, C82, a modulator of Wnt/β-catenin signaling, has been developed. When used in conjunction with nilotinib, C82 effectively curtailed the proliferation of primary CML cells from patients, both with and without the *BCR–ABL1* mutation. The use of siRNA to suppress β-catenin expression reinstated the sensitivity of these cells to nilotinib in patients harboring the *BCR–ABL1* T315I mutation. Impressively, combining C82 with nilotinib significantly boosted the survival rate of mice in CML in vivo models. These findings underscore the potential of a dual inhibition strategy targeting β-catenin and BCR–ABL1 to prevent and counteract TKI therapy resistance [70].

In the quest for new targeted CML therapies, the oncogenic *DUXAP10* pseudogene has been identified as a key player in CML progression, through its activating of cell proliferation, disruption of cell cycle control, and inhibition of apoptosis via downregulation of *phosphatase and tensin homolog deleted on chromosome 10* (*PTEN*) expression. PTEN, a crucial negative regulator of the PI3K/AKT/mTOR signaling pathway, is known as an “anti-cancer protein”. Knockdown of *DUXAP10* by siRNA led to significant reduction in cell proliferation, cell cycle arrest, and increased apoptosis [71]. Additionally, the *Tripartite Motif-Containing 22* (*TRIM22*) gene emerged as a viable target for siRNA-mediated knockdown, with its suppression resulting in reduced cell proliferation and enhanced apoptosis through the inhibition of the PI3K/AKT/mTOR signaling pathway and altered levels of CDK4, cyclin D1, P70S6K, and P53 in K562 cells [72].

In 2007, a clinical case involving siRNA therapy in a patient with recurrent Ph+ CML, resistant to imatinib (harboring the Y253F mutation), and undergoing chemotherapy post-allogeneic HSCT, was documented. The siRNA therapy was administered alongside ongoing treatment with imatinib and cytarabine (ara-C). The siRNA was delivered intravenously as a dispersed lipid solution using a liposomal transfection agent. The patients received three injections, which were well tolerated and resulted in no significant adverse effects. The most significant reduction in *BCR–ABL1* gene expression followed the first dose, plummeting from 6.6% to 0.053% (based on the ratio of *BCR–ABL1* to control gene expression on an international scale) within nine days. However, expression levels began to rise again, reaching 16.1% twenty-two days post-administration. Although subsequent injections, even at increased siRNA doses, did not replicate the effect, ex vivo siRNA transfections on leukemic cells isolated from the patient pre- and post-treatment revealed diminished responsiveness following the last treatment. This could indicate resistance to the combined imatinib and cytosar therapy or insufficient therapeutic intensity. Despite mixed outcomes, this study highlights the potential feasibility and therapeutic promise of siRNA in CML treatment using non-viral delivery methods [73].

It is important to note that, despite the extensive research on CML models in vitro and the clinical application of BCR–ABL1 siRNA as outlined previously, the inaugural study utilizing BCR–ABL1 siRNA in vivo within a xenograft mouse model of CML was only published in 2018 [60]. The study employed lipopolymer nanoparticles composed of α-linolenic acid and low molecular weight polyethyleneimine as a vehicle for drug delivery. The drug was administered both subcutaneously near the tumor site and intraperitoneally. Although a reduction in tumor growth rate was noted, a statistically significant diminution in tumor size, in comparison to the control group, was only observed on day 7 out of a 14-day period when the drug was administered subcutaneously; no significant effect was reported with intraperitoneal administration. In contrast, TKIs have demonstrated far greater efficacy in suppressing tumor growth in preclinical in vivo studies. This discrepancy highlights the differing mechanisms of action between siRNAs and small molecules on *BCR–ABL1*-expressing cells, suggesting that the intracellular delivery system utilized may not have been sufficiently effective in vivo. Furthermore, the study of siRNA-based therapeutics necessitates a more relevant in vivo CML model, specifically transplantation of cells derived from CML patients into immunodeficient NOD/SCID/gamma-chain mice, to conduct a thorough analysis of systemic injection toxicity of siRNAs, both alone and in conjunction with transfection agents, and to ascertain their therapeutic anticancer efficacy [60].

Historically, the absence of an efficient and safe siRNA delivery system stood as the principal barrier to the clinical adoption of RNA interference–based therapies. In 2018, the FDA approved the first drug of this nature, Patisiran, developed by Alnylam for treating a rare hereditary disorder known as transthyretin amyloidosis (hATTR) with polyneuropathy. Comprising siRNA molecules affiliated with lipid nanoparticles (LNPs), Patisiran accomplishes effective delivery of siRNA to the liver upon intravenous application. Nonetheless, the challenge of deploying LNPs for siRNA delivery, especially to other critical organs like the hematopoietic system, remains unresolved. A groundbreaking study in 2019 introduced a novel approach that harnesses LNPs and a microfluidics system for encapsulating siRNAs into particles aimed at the *BCR–ABL1* gene in hematopoietic cells. These LNP-siRNA complexes exhibited near-perfect efficacy in both in vitro and in vivo experiments, including delivery and suppression of the target gene in primary cell cultures and a xenograft mouse model of CML via intravenous administration. The promising outcomes of this study illuminate the potential of siRNA-based antisense therapy to curb leukemic cell proliferation, heralding new avenues for CML treatment [74].

## 4. Conclusions

Chronic myeloid leukemia stands as a distinct cancer type, especially when considering its molecular genetic underpinnings. Central to this disease is a specific mutation that give rise to the *BCR–ABL1* chimeric oncogene, a factor both necessary and sufficient for the onset of the malignancy. Before the advent of targeted treatment, allogeneic hematopoietic stem cell transplantation (allo-HSCT) represented the only potentially curative treatment option in CML [75]. However, the introduction of TKIs has dramatically transformed the prognosis and quality of life for patients with CML. Presently, TKIs are a lifelong therapy for many patients. A potential new goal of CML therapy is treatment-free remission (TFR), driven by the long-term survival outcomes seen with the use of TKIs. TFR is defined as the ability to maintain minimal residual disease at undetectable or stable low levels after discontinuing TKIs. Many patients who have an optimal response can achieve a life expectancy comparable to that of the general population. Although TKI treatment is beneficial, its prolonged use can come with significant cost and burden to patients. Recently, studies have shown that in a select group of patients with chronic-phase CML, TKIs can be safely discontinued [76,77]. Current clinical trials concentrate on identifying criteria for safe and effective discontinuation of TKIs and expanding the pool of patients that successfully achieve TFR [78].

The exploration and development of a novel class of RNAi-based therapeutics, capable of specifically targeting *BCR–ABL1* gene expression, shows promise in mitigating resistance to tyrosine kinase inhibitors and providing an additional treatment pathway for CML patients. Furthermore, the synergistic use of BCR–ABL1 inhibitors alongside RNAi therapeutics aimed at the *BCR–ABL1* transcript could facilitate a swifter molecular response, enhancing the likelihood of achieving TFR and subsequently, therapy discontinuation therapy. The achievement of TFR is becoming increasingly achievable in CML patients; however, in the near future, the use of RNAi therapeutics may further improve the path to TFR and widen the TFR population in clinical practice.

One of the principal challenges in the broader adoption of RNAi-based therapeutic strategies lies in devising efficient delivery mechanisms for these drugs to targeted cells. Consequently, the rational design of non-viral nucleic acid delivery systems to address the current limitations in siRNA delivery efficiency remains an imperative task for researchers aiming to bring siRNA-based gene therapy to clinical application.

## Figures and Tables

**Table 1:** BCR–ABL1 targeted therapeutics. Information is presented based on https://www.fda.gov/ (accessed on 24 May 2024) and https://classic.clinicaltrials.gov/ (accessed on 24 May 2024).

Drug	Pharmacological Strategy for Inhibiting the BCR–ABL1	Mechanism of Action	Activity against T315I Mutations	Clinical Status
First-line treatment				
Imatinib (Gleevec; Novartis)	ATP-competitive inhibitor	first generation TKI	no	FDA approved
Dasatinib (Sprycel; Bristol Myers Squibb)	ATP-competitive inhibitor	second-generation TKI	no	FDA approved
Nilotinib (Tasigna; Novartis)	ATP-competitive inhibitor	second-generation TKI	no	FDA approved
Bosutinib (Bosulif; Pfizer)	ATP-competitive inhibitor	second-generation TKI	no	FDA approved
Ponatinib (Iclusig; Takeda)	ATP-competitive inhibitors	third-generation TKI	yes	FDA approved
Asciminib (Scemblix; Novartis)	Allosteric inhibitor	targeting the ABL myristoyl pocket (STAMP)	yes	FDA approved
Emerging Therapies				
Radotinib	ATP-competitive inhibitors	second-generation TKI	no	NCT03722420
Flumatinib	ATP-competitive inhibitors	second-generation TKI	no	NCT02204644
Olverembatinib (HQP1351)	ATP-competitive inhibitors	third-generation TKI	yes	NCT05594758
Vodobatinib (K0706)	ATP-competitive inhibitors	third-generation TKI	yes	NCT02629692
